# Comparison of muscle activity in the empty-can and full-can testing positions using ^18^ F-FDG PET/CT

**DOI:** 10.1186/s13018-014-0085-4

**Published:** 2014-10-01

**Authors:** Choon-Key Lee, Eiji Itoi, Seong-Jang Kim, Su-Chan Lee, Kuen-Tak Suh

**Affiliations:** Department of Orthopaedic Surgery, Pusan Himchan Hospital, 275-7, Suan-dong, Dongnae-gu, Pusan, 607-824 Korea; Department of Orthopaedic Surgery, Tohoku University School of Medicine, 1-1 Seiryomachi, Aoba-ku, Sendai, 980-8574 Japan; Department of Nuclear Medicine, Pusan National University School of Medicine, 10-1, Ami-dong, Seo-gu, Pusan, 602-739 Korea; Department of Orthopedic Surgery, Himchan Hospital, 404-3, Mok-dong, Yangcheon-gu, Seoul, 158-806 Korea; Department of Orthopaedic Surgery, Pusan National University Yangsan Hospital, Beomeo-ri, Mulgeum-eup, Yangsan-si, Gyeongsangnam-do, 626-770 Korea

**Keywords:** Fluoro-D-glucose, Positron emission tomography, Empty-can test, Full-can test, Muscle activity, Rotator cuff

## Abstract

**Background:**

There has been much controversy over specific tests for diagnosis of supraspinatus tendon tear. The aim of this study was to evaluate the metabolic activity of the deltoid and rotator cuff muscles while maintaining the full-can and empty-can testing positions using 2-deoxy-2-[^18^ F]fluoro-D-glucose (^18^ F-FDG) positron emission tomography (PET)/computed tomography (CT).

**Methods:**

Ten healthy volunteers without shoulder pain or diabetes mellitus participated in this study. Following FDG injection, both arms were maintained in either the empty-can or full-can position for 10 min. PET/CT was performed 40 min after injection. Maximum standardized uptake values (SUVs) were measured in the deltoid and rotator cuff muscles on axial PET images.

**Results:**

The middle deltoid exhibited the most significant increase in muscle activity at both testing positions. Additionally, a significant increase in muscle activity was observed in the middle deltoid compared with the supraspinatus (*P* < 0.05) in the empty-can testing position. SUVs of the middle deltoid, supraspinatus, and subscapularis showed a significant increase in the empty-can testing position compared with the full-can testing position (*P* < 0.05).

**Conclusions:**

Significantly increased activity of the supraspinatus in conjunction with the middle deltoid and subscapularis after empty-can testing may result in decreased specificity of the empty-can test in detecting isolated supraspinatus activity. The full-can test, however, may be used to test the function of the supraspinatus with the least amount of surrounding middle deltoid and subscapularis activity.

## Background

The empty-can test, which Jobe and Moynes initially described in 1982, is one of the most useful methods for identifying supraspinatus tears on physical examination [1]. More recently, however, Kelly et al. reported that the full-can test for assessment of supraspinatus function at 90° elevation in the scapular plane and 45° of external rotation had similar electromyographic (EMG) activity compared with the empty-can test, but that it provoked less pain [2]. The full-can testing position was also recommended for manual muscle testing and rehabilitation of the supraspinatus muscle. However, several EMG studies have examined the effectiveness of strengthening exercises using the full-can versus empty-can positions and have yielded inconsistent results [3-5]. This may be due to several limitations of EMG, such as the fact that the intramuscular fine needle electrodes used in EMG reflect the activity of only a small number of muscle fibers and sometimes migrate during exercise [6,7].

2-deoxy-2-[^18^ F]fluoro-D-glucose (FDG) positron emission tomography (PET) has recently been used for assessment of skeletal muscle activity [8-11]. Little uptake of FDG, a radioactive deoxy-analog of glucose, occurs in resting muscle. However, FDG does accumulate in exercising muscles [12]. The basis of FDG PET imaging is that following intravenous injection of FDG, exercising muscle first utilizes available glucose and subsequently consumes circulating FDG. The FDG is then phosphorylated, converted into FDG-6-phosphate, and entrapped in the intracellular space. Unlike glucose, FDG-6-phosphate does not participate further in the usual glycolytic pathway and instead accumulates within exercising muscle tissue [13,14]. Radioactive ^18^ F can be detected by a PET scanner; thus, FDG that has accumulated in the muscle cell can be used as an indicator of muscle activity. Shinozaki et al. reported that FDG uptake in the supraspinatus muscle following a cuff exercise protocol decreased on the ruptured side compared with the intact contralateral side, demonstrating a close relationship between FDG accumulation and muscle activity [10]. The purpose of this study was to evaluate and compare muscle activity of the rotator cuff and deltoid at the full-can and empty-can testing positions. To the best of our knowledge, this is the first report to compare activity of the rotator cuff and deltoid muscles using PET/CT while maintaining the arm in the empty-can and full-can testing positions. Our results may provide clinicians with valuable information regarding which muscles are best evaluated on physical examination in the full-can or empty-can testing positions.

## Methods

### Participants

Ten healthy male subjects aged from 27–34 years (mean 29.8 ± 2.7) volunteered to participate in this study. Subjects who had undergone previous shoulder surgery or those with diabetes mellitus were excluded from the study. This study protocol was approved by the Hospital Ethic Committee of Himchan Hospital Health System (No.: 10-2010, Date: April 28, 2010) and a signed consent form was obtained from each subject.

### Testing procedures and PET/CT imaging

**Figure 1:**
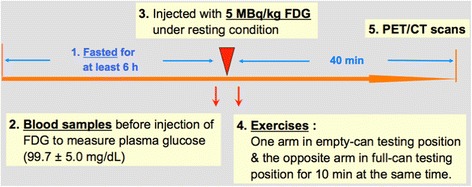
**Timeline for the testing procedure.**

**Figure 2:**
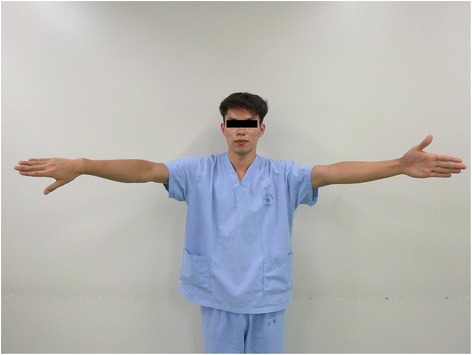
**Empty-can and full-can testing positions.** The volunteers were instructed to hold their arm in either the empty-can (90° elevation in the scapular plane with full internal rotation) or full-can position (90° elevation in the scapular plane with full external rotation) for 10 min.

All subjects fasted for at least 6 h prior to the PET/CT study. After intravenous injection of FDG (5 MBq/kg), they were instructed to maintain their arm in either the empty-can or full-can position for 10 min (Figure 1). The full-can position is described as holding the arm at 90° elevation in the scapular plane (30° anterior to the frontal plane) with full external rotation while the empty can position is achieved by holding the arm at 90° elevation in the scapular plane with full internal rotation (Figure 2). Subjects were randomly distributed into two groups, one of which performed the empty can test on the right arm and the full can test on the left arm, and vice versa in the second group. To ensure proper body positioning without compensatory movements during data collection, all tests were performed in the presence of one of the investigators (CL). Before injection of FDG, intravenous blood samples were drawn for measurement of plasma glucose concentration, which was within normal limits for all subjects (mean 99.7 ± 5.0 mg/dL). Forty minutes after an intravenous injection of FDG, PET/CT scans were performed from neck to trunk with a germanium oxyorthosilicate full-ring PET scanner and a dual slice helical CT scanner (Gemini, Philips, Milpitas, CA, USA).

### Image analysis

Regions of interest (ROIs) were defined as the area over the deltoid (anterior, middle, and posterior), supraspinatus (SSp), infraspinatus (ISp), subscapularis (SSc), and upper thoracic spine muscles (control) in axial slices extending from the acromion to the inferior end of the scapula. These muscles were easily recognized on axial CT images using scapular bony landmarks including the acromion and scapular spine as reference points. The maximum standardized uptake value (SUV) was measured in all of the axial slices from ROIs for quantification of muscle activity after the empty-can and full-can tests. SUVs were generated using attenuation-corrected images based on the amount of FDG injected, the patient’s body weight, and cross-calibration factors between PET and the dose calibrator. When FDG uptake was observed in ROIs, the hypermetabolic muscles were identified and recorded. SUVs of PET and PET/CT images were evaluated by a nuclear radiologist that was blinded to the test groups.

### Statistical analysis

All experimental data were presented as the mean and standard deviation of the mean (SD). A commercial software package (GraphPad Prism version 5.0 for Macintosh, GraphPad Software Inc, San Diego, CA) was used to perform statistical analysis of the data. Two-way repeated-measures ANOVA followed by Tukey’s *post hoc* tests were used for multiple comparisons of the mean maximum SUVs of each muscle tested. A *P* value < 0.05 was regarded as a significant difference between means.

## Results

**Figure 3:**
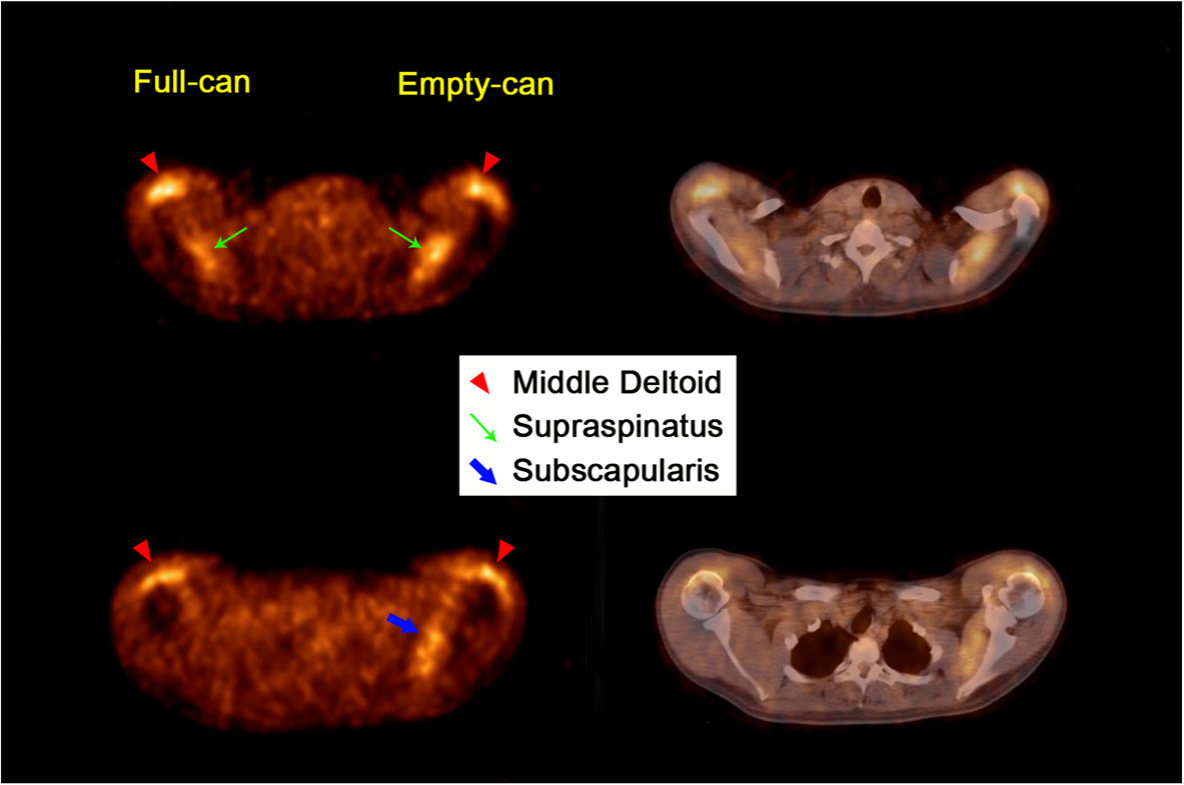
**Axial views of PET/CT.** The PET/CT images of one case demonstrated increased muscle activity of the middle deltoid, supraspinatus, and subscapularis after maintaining the arm in the empty-can versus the full-can testing position.

Mean maximum SUVs of the deltoid and rotator cuff muscles in the individual subjects at the full-can and empty-can testing positions are shown in Table 1. All ten subjects showed FDG uptake in the middle deltoid and supraspinatus muscles in both the empty-can and full-can testing positions (Figure 3). However, there was no FDG uptake in the posterior deltoid at the full-can testing position. FDG uptake was seen in the posterior deltoid in only two subjects in the empty-can testing position. And there was no FDG uptake in the upper thoracic spine muscles in both positions. The subscapularis muscle showed increased activity in nine subjects in the empty-can testing position.

**Table 1:** **The mean maximum SUV of the deltoid and rotator cuff muscles in individual subjects in the full-can and empty-can testing positions**

**Cases**	**Deltoid**						**Supraspinatus**		**Infraspinatus**		**Subscapularis**		**Thoracic spine**	
	**Anterior**		**Middle**		**Posterior**									
	**Full**	**Empty**	**Full**	**Empty**	**Full**	**Empty**	**Full**	**Empty**	**Full**	**Empty**	**Full**	**Empty**	**Full**	**Empty**
*1*	0.83	0.85	1.47	2.60	0.51	0.54	1.41	1.88	0.77	0.73	0.84	1.57	0.52	0.51
*2*	0.99	0.98	2.70	2.66	0.68	0.62	1.88	2.55	0.69	0.62	0.87	1.42	0.61	0.58
*3*	0.68	0.70	1.94	2.05	0.36	0.42	2.11	1.82	0.45	0.59	0.85	1.25	0.42	0.48
*4*	0.65	0.66	1.02	1.92	0.43	0.48	1.50	1.64	0.86	0.57	0.64	0.74	0.45	0.49
*5*	0.89	0.72	2.13	2.31	0.41	1.84	1.81	1.18	0.95	0.89	0.85	1.29	0.42	0.48
*6*	0.81	0.86	1.47	1.88	0.56	0.91	1.59	2.05	0.76	0.72	1.14	1.39	0.51	0.55
*7*	0.89	0.87	2.78	2.82	0.61	0.51	1.24	1.87	0.85	0.83	0.92	2.20	0.59	0.52
*8*	0.61	0.86	1.49	1.92	0.52	0.55	1.33	1.54	0.83	0.69	0.80	1.44	0.55	0.59
*9*	0.58	0.83	1.55	2.67	0.45	0.49	1.38	2.96	0.60	0.72	0.75	0.97	0.49	0.53
*10*	0.87	0.99	1.98	2.98	0.63	0.49	1.25	1.86	0.70	0.61	0.81	0.85	0.59	0.55
*Ave*	0.78	0.83	1.85	2.38	0.52	0.69	1.55	1.94	0.75	0.70	0.85	1.31	0.52	0.53
*SD*	0.14	0.11	0.57	0.42	0.10	0.43	0.29	0.50	0.14	0.10	0.13	0.42	0.07	0.04

### Full-can testing position

**Figure 4:**
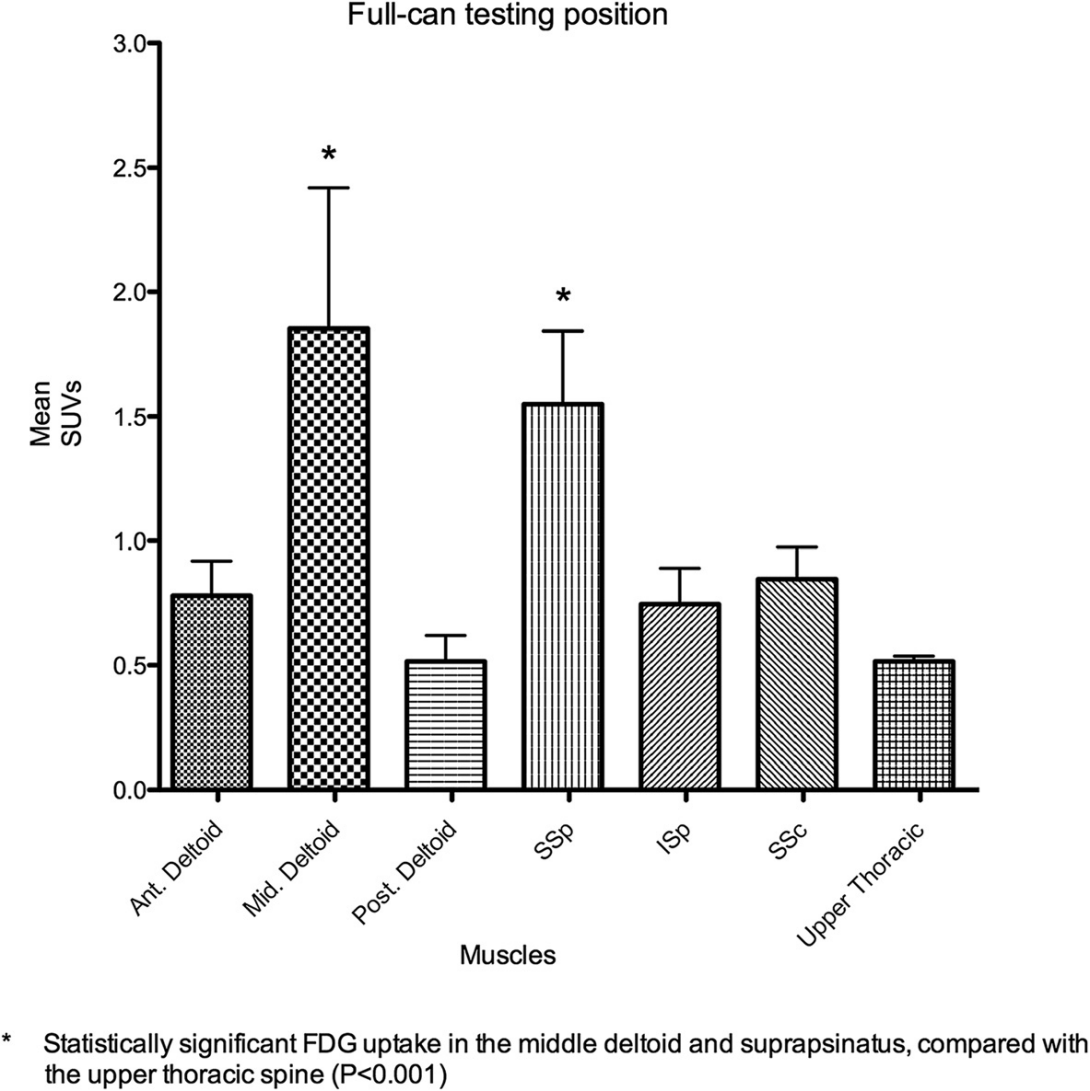
**Comparison of mean maximum SUVs of the muscles in the full-can testing position.** The middle deltoid showed the greatest increase of muscular activities among the six muscles in this position. The middle deltoid and supraspinatus showed significantly greater muscular activity than the anterior and posterior deltoid, infraspinatus, and subscapularis (*P* < 0.001).

Among the six muscles tested, the middle deltoid muscle showed the highest FDG uptake, followed by the supraspinatus muscle. *Post hoc* analysis of the full-can testing position revealed significantly greater muscle activity of the middle deltoid and supraspinatus muscles compared with the anterior deltoid, posterior deltoid, infraspinatus, subscapularis, and upper thoracic spine muscles (*P* < 0.001). However, there was no statistical difference between the middle deltoid and supraspinatus muscles (Figure 4).

### Empty-can testing position

**Figure 5:**
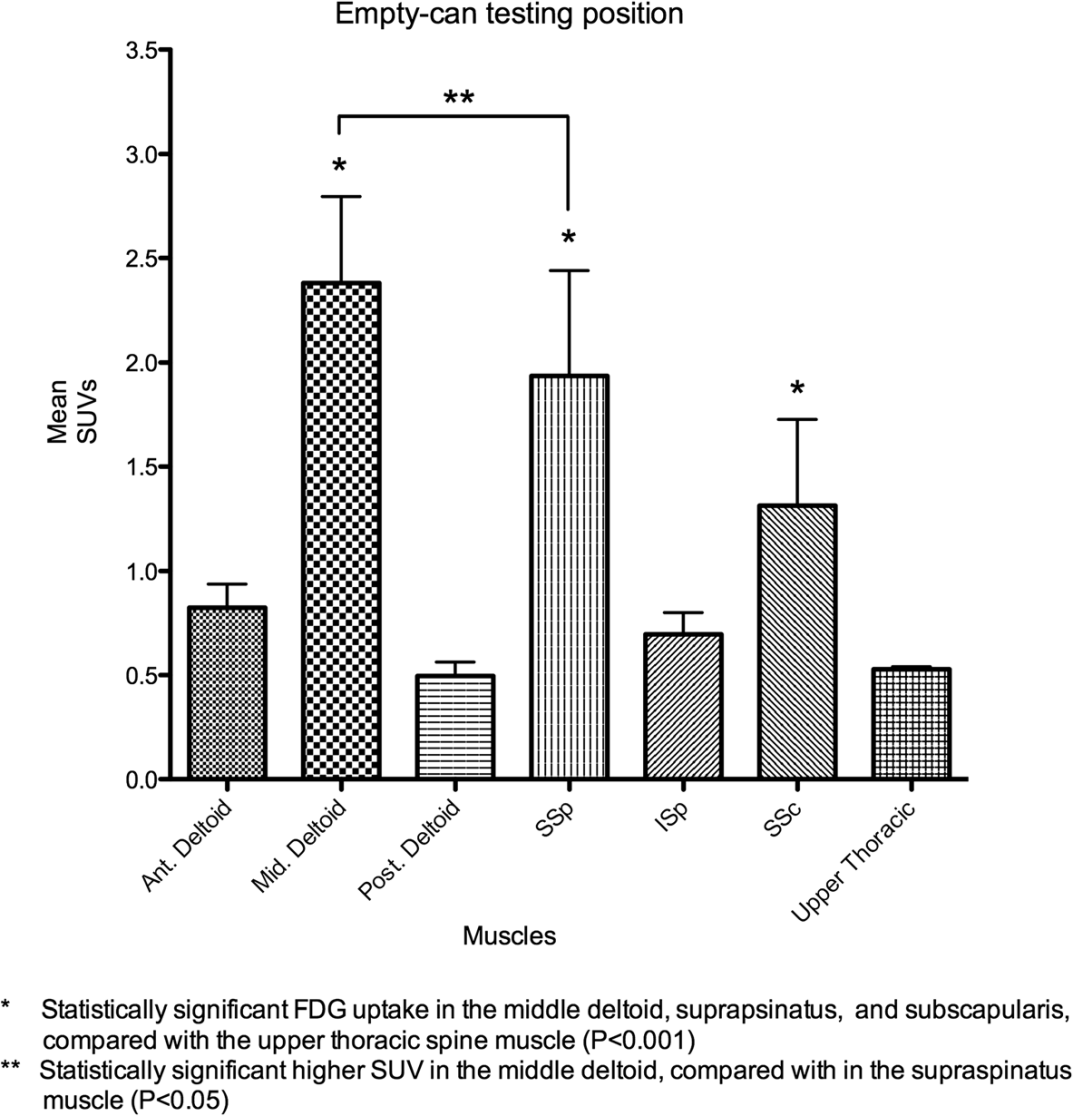
**Comparison of mean maximum SUVs of the muscles in the empty-can testing position.** The middle deltoid exhibited the highest FDG uptake among the six muscles in this position. The middle deltoid, supraspinatus, and subscapularis showed significantly increased muscular activity than the anterior deltoid, posterior deltoid, and infraspinatus. Muscular activity showed a significant increase in the middle deltoid, compared with the supraspinatus (*P* < 0.05).

The middle deltoid muscle exhibited the highest FDG uptake among the six muscles, followed by the supraspinatus and subscapularis muscle. *Post hoc* analysis of the empty-can position showed significantly increased muscle activity of the middle deltoid and supraspinatus muscles, compared with the anterior deltoid, posterior deltoid, infraspinatus, and upper thoracic spine muscles (*P* < 0.001). A statistically significant increase in muscle activity was observed in the middle deltoid muscle compared with the supraspinatus muscle (*P* < 0.05). Muscle activity was significantly greater in the subscapularis muscle than in the anterior deltoid (*P* < 0.05), posterior deltoid (*P* < 0.001), infraspinatus (*P* < 0.001), and upper thoracic muscles (*P* < 0.001) (Figure 5).

### Comparison between the full-can and empty-can testing positions

**Figure 6:**
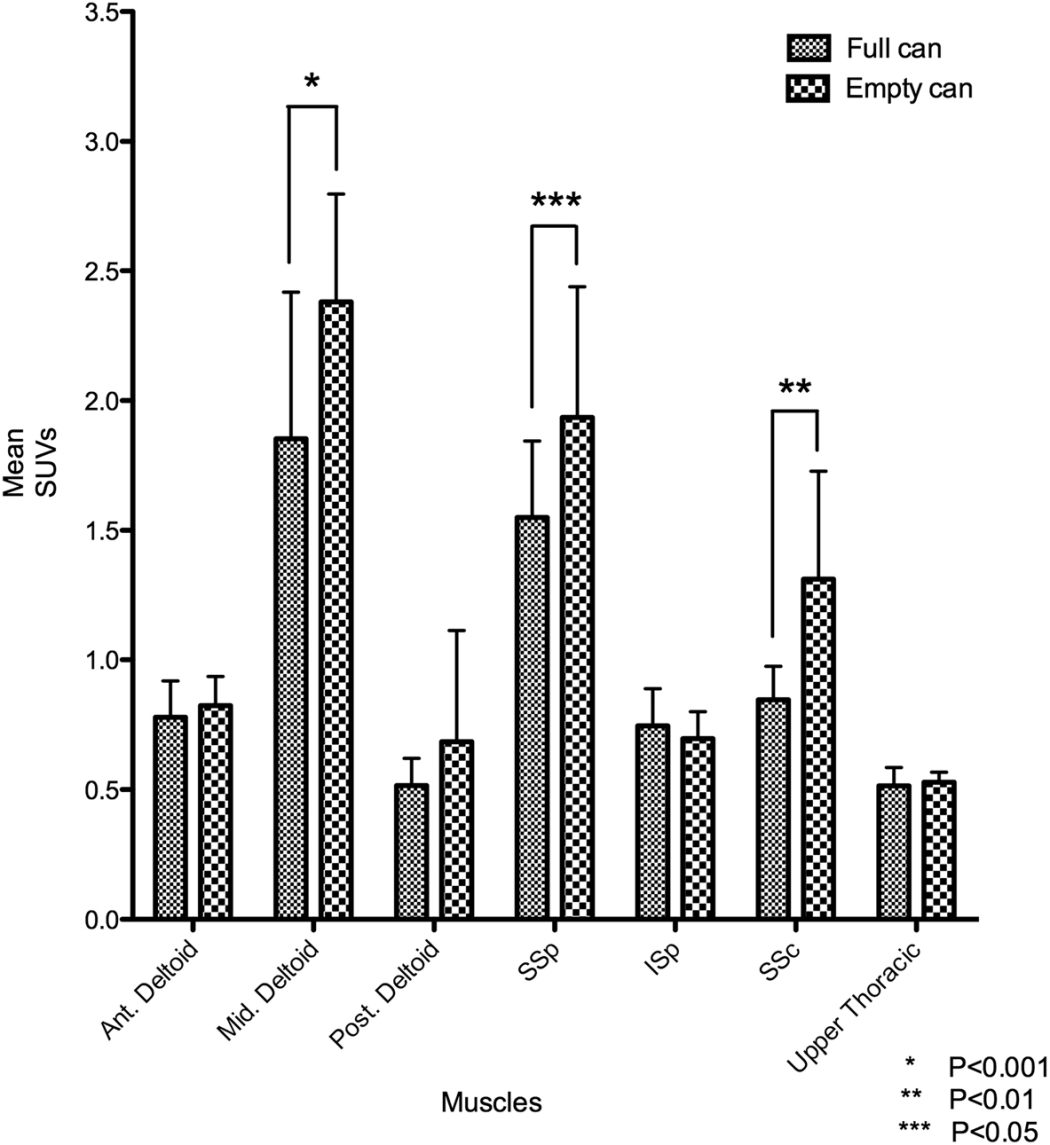
**Comparison of mean maximum SUVs of each muscle in the full-can versus empty-can testing positions.** Statistically significant increases of the mean maximum SUVs were observed in the middle deltoid (*P* < 0.001), supraspinatus (*P* < 0.01), and subscapularis (*P* < 0.05) during the empty-can testing position, compared with during the full-can testing position.

Statistically significant increases in SUVs were observed in the middle deltoid (*P* < 0.001), supraspinatus (*P* < 0.01), and subscapularis muscles (*P* < 0.05) in the empty-can compared with the full-can testing position (Figure 6). In calculating the average ratio of the mean maximum SUV in the each muscle to the total mean maximum SUVs of all six muscles, we found no statistically significant differences between the full-can and empty-can positions, although the average ratio for the infraspinatus and subscapularis muscles were greater in the full-can position and empty-can position respectively (Table 2).

**Table 2:** **Average ratio of the mean maximum SUV in each muscle to the total mean maximum SUVs of all six muscles**

**Ratio (%)**	**Deltoid**			**SSp**	**ISp**	**SSc**
	**Anterior**	**Middle**	**Posterior**			
Full-can position	12%	29%	8%	25%	12%	14%
Empty-can position	11%	30%	9%	25%	9%	17%

No significant difference between full-can and empty can position (*P* > 0.05).

## Discussion

The supraspinatus and the middle deltoid muscles are generally thought to be the two most important muscles in executing arm elevation in the scapular plane [15-17]. In the full-can testing position, our study found that the middle deltoid and supraspinatus muscles showed significantly greater muscle activity than the anterior deltoid, posterior deltoid, infraspinatus, subscapularis, and upper thoracic muscles, indicating that these are the most important muscles for 90° scaption with the thumb up position (Figure 3). The middle deltoid showed higher, though not statistically increased, muscle activity than the supraspinatus in this position. Our results differed from those of Reinold et al. and Takeda et al., who reported that the supraspinatus muscle showed greater muscle activity than the middle deltoid muscle [18,19]. Although it is controversial whether the middle deltoid or the supraspinatus is more active, we propose that the full-can test is more specific to the supraspinatus muscle than the deltoid or other rotator cuff muscles because the deltoid usually maintains a normal function in the rotator cuff tear.

In our study, the empty-can testing position (Figure 4) showed significantly increased muscle activity of the middle deltoid, supraspinatus, and subscapularis muscles, compared with the anterior deltoid, posterior deltoid, infraspinatus, and upper thoracic muscles. In addition, significantly higher muscle activity was observed in the middle deltoid muscle compared with the supraspinatus muscle. Townsend et al. and Reinold et al. also found that the middle deltoid muscle showed greater EMG activity than the supraspinatus muscle during the empty-can exercise; however, statistical significance was not noted in their data [5,18]. Rowlands et al. reported that EMG activity from the middle deltoid muscle showed a markedly higher amplitude than that of the supraspinatus muscle, and that supraspinatus activity could not be isolated during the empty-can test [4]. Our data support these previous findings, and additionally suggest that increased metabolic activity of the subscapularis muscle in our study can make the empty-can exercise and test less specific to the supraspinatus muscle than the full-can exercise and test.

Comparison of muscle activity between the full-can and empty-can positions in our study (Figure 5) showed significantly higher activity in the middle deltoid (*P* < 0.001), supraspinatus (*P* < 0.01), and subscapularis muscles (*P* < 0.05) during empty-can testing versus full-can testing. These results are similar to those reported by Townsend et al., who demonstrated a 10% increase in supraspinatus activity during performance of the empty-can exercise compared with the full-can exercise, and thus concluded that this position best isolated the supraspinatus [5]. Unlike the current study, several papers have failed to demonstrate a difference in supraspinatus activity between the empty-can and full-can exercise; however, more activity in surrounding muscles was observed during the empty-can exercise [2,6,18,19]. We propose that the full-can test is a more effective way to examine the supraspinatus muscle than the empty-can test since the full-can testing position had less activity in the surrounding muscles such as the subscapularis and middle deltoid. Interestingly, the average ratio of the mean maximum SUV in the deltoid and supraspinatus to total mean maximum SUVs of all six muscles showed almost the same results between the full-can and empty-can positions. However, the average ratio in the subscapularis was higher in the empty-can position than the full-can position (Table 2).

The primary difference in strength requirements of the middle deltoid, supraspinatus, and subscapularis between the full-can and empty-can tests is due to the position of the arm. We propose that extra force is needed to keep the arm internally rotated during empty-can testing, particularly in the middle deltoid and supraspinatus which act as the primary elevators. This may be one of the reasons why the middle deltoid showed significantly higher muscle activity than the supraspinatus at 90° of scaption in the thumb down position. Reinold et al. suggested that increased activity of the middle deltoid during the empty-can exercise relative to the full-can exercise may lead to a superiorly directed shear force of humeral head, which can cause pain or discomfort [18]. Additionally, Itoi et al. showed that both the empty-can and full-can tests are equivalent with regard to accurately diagnosing muscle weakness; however, when considering pain provocation, the full-can test may be more beneficial in the clinical setting [20]. Otis et al. showed that the upper subscapularis had a significant role in arm elevation in the scapular plane [15]. In our study, the subscapularis worked only as an internal rotator during these two static exercises, as there was no significant increase in FDG uptake in the subscapularis during the full-can test. Gerber and Krushell reported that among 16 cases of isolated subscapularis rupture, seven patients showed weakness in elevation in the scapular plane, indicating a positive empty-can test [21]. These data suggest that subscapularis pathology can induce pain or weakness during the empty-can test.

Our PET study has certain advantages over conventional intramuscular wire EMG studies [9]. Using PET, we were able to noninvasively obtain anatomic information about the location of the active muscles in the human body after specific exercises. In addition, we were able to compare metabolic activity of the involved muscles using the degree of FDG uptake into the exercising muscles.

The present study has several limitations to consider. First, the number of patients enrolled in our study could be too small to draw meaningful conclusions. Second, SUVs in the upper thoracic spine muscles were used as baseline data but may have also been activated by empty-can and full-can testing. It should be noted, however, that the muscle activity on PET is determined by both color change as well as SUVs in the images generated. There were no color changes observed in the upper thoracic spine muscles in our study. Third, PET requires exposing the enrolled patients to radiation, although the amount of FDG injected into each subject was similar to the dose used in conventional and diagnostic studies [10]. Finally, PET does not reflect a real time contraction of each muscle, and as a result our data may not directly correlate with empty-can or full-can testing in a clinical setting. While in our study the exercises were done for a total of 10 min, in a real time scenario these muscles contract instantly against the load applied by the examiner. This raises the possibility of secondary recruitment occurring as muscles become fatigued, which may affect the relative contribution of the muscles. In addition, we do not know the duration of exercise required for FDG uptake to become apparent on PET scan. The authors supposed that the exercises in our study are the closest method of exercises which demonstrated muscle activities of the empty-can and full-can tests with PET scan without any unnecessary movements affecting the results. However, the exercises in this study cannot represent the actual empty-can and full-can tests, which may affect the conclusion. Although the strong suggestion does not mention from this study, current study showed baseline data of the muscle activity elevating with two positions using the reliable procedure.

## Conclusions

The empty-can testing position requires significantly increased activity of the supraspinatus in conjunction with the middle deltoid and subscapularis muscles, which may make this test less specific for detecting weakness in the supraspinatus muscle in the clinical setting. Our results suggest, however, that the full-can test may be used to assess the function of the supraspinatus with the least amount of surrounding middle deltoid and subscapularis activity.
